# Cesium Reduction of a Lithium Diamidochloroberyllate

**DOI:** 10.1021/acs.organomet.3c00519

**Published:** 2024-01-24

**Authors:** Kyle G. Pearce, Michael S. Hill, Mary F. Mahon

**Affiliations:** Department of Chemistry, University of Bath, Claverton Down, Bath BA2 7AY, U.K.

## Abstract

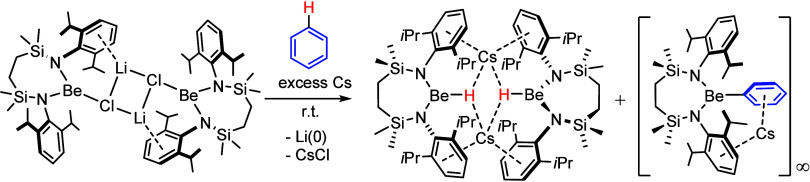

Room temperature reaction of elemental cesium with the dimeric lithium chloroberyllate [{SiN^Dipp^}BeClLi]_2_ [{SiN^Dipp^} = {CH_2_SiMe_2_N(Dipp)}_2_, where Dipp = 2,6-di-isopropylphenyl, in C_6_D_6_ results in activation of the arene solvent. Although, in contrast to earlier observations of lithium and sodium metal reduction, the generation of a mooted cesium phenylberyllate could not be confirmed, this process corroborates a previous hypothesis that such beryllium-centered solvent activation also necessitates the formation of hydridoberyllium species. These observations are further borne out by the study of an analogous reaction performed in toluene, in which case the proposed generation of formally low oxidation state beryllium radical anion intermediates induces activation of a toluene *sp*^3^ C–H bond and the isolation of the polymeric cesium benzylberyllate, [Cs({SiN^Dipp^}BeCH_2_C_6_H_5_)]_∞_.

## Introduction

Following a period of relative dormancy,^1^ the molecular chemistry of beryllium has experienced something of a renaissance during the past decade. While the solution behavior and toxicity of organo- and inorganic beryllium(II) compounds continues to provide a significant conventional focus,^2−9^ noteworthy effort has also been applied to the pursuit of lower oxidation state Be species.^10−13^ In this latter regard, the groups of Braunschweig and Gilliard have succeeded in the isolation of several cyclic alkyl(amino)carbene (CAAC)-supported compounds in which formal Be(0) or Be(I) oxidation states may be ascribed to an isolated beryllium center.^14−17^ Notably, and very recently, the long sought^18,19^ and predicted^20,21^ isolation of a Be–Be bonded species has been realized through Aldridge and co-workers’ isolation of CpBeBeCp.^22,23^

**Figure 1:**
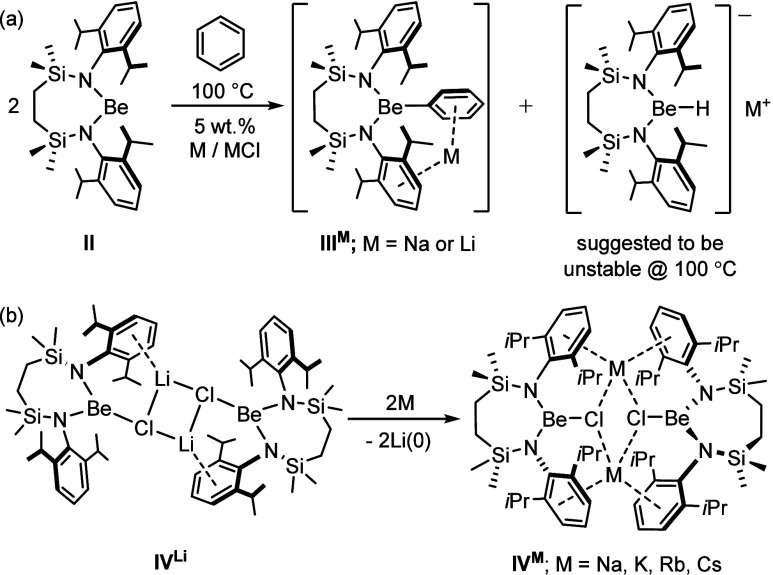
(a) Beryllium-centered benzene activation induced by attempted reduction of **II**; (b) reduction of Li^+^ resulting from reaction of **IV**^**Li**^ by Na, K, Rb, or Cs metal.

Inspired by Jones and co-workers’ now extensive development of Mg–Mg bonded species,^10,18,24−27^ we have recently reported the bimetallic Mg(I)Na(I) derivative, [{SiN^Dipp^}MgNa]_2_ (**I**; [{SiN^Dipp^} = {CH_2_SiMe_2_N(Dipp)}_2_, where Dipp = 2,6-di-isopropylphenyl).^28−30^ Attempting to apply a similar heterobimetallic motif to magnesium’s lighter congener, we have observed that Li or Na reduction of the chelated beryllium dianilide, [{SiN^Dipp^}Be] (**II**), results in C–H activation of the benzene solvent and isolation of phenylberyllate species, [M({SiN^Dipp^}BePh)] (**III**^**M**^, where M = Li or Na; Figure 1a).^31^ Although no definitive spectroscopic or structural evidence could be obtained, we tentatively suggested that this chemistry invoked the generation of transient Be(I) radical anions, onward reaction of which with the solvent provided a mixture of **III**^**M**^ and a presumed hydridoberyllate species, [M({SiN^Dipp^}BeH)], which was unstable under the applied reaction conditions (100 °C).

In an extension to this research, we have very recently reported that reduction of the dimeric lithium chloroberyllate (**IV**^**Li**^) with either sodium, potassium, or rubidium metal provides no discernible beryllium reduction but results in exclusive replacement of the lithium cations to provide, [{SiN^Dipp^}BeClM]_2_ (**IVM**, where M = Na, K, Rb; Figure 1b).^32^ Although similar treatment of **IV**^**Li**^ with Cs metal also enabled the isolation of a cesium analogue, **IV**^**Cs**^, this reaction required immediate low temperature workup to suppress the formation of further products. Prompted by the supposition that this onward reactivity invokes subsequent beryllium-centered reduction of **IV**^**Cs**^ by the most electropositive available group 1 metal, we now report our further observations of this reaction system.

## Experimental Section

### General Considerations

**CAUTION:** Beryllium and its compounds are extremely toxic. Suitable precautions (e.g., use of protective clothing, a breathing apparatus, and a well-ventilated fume cupboard) should be taken for all manipulations involving these species. Our general policy when handling beryllium compounds and reagents is to limit the potential for ambient proliferation by working on a microscale.

All manipulations were carried out using standard Schlenk line and glovebox techniques under an inert atmosphere of argon. NMR experiments were conducted in J. Young tap NMR tubes prepared in a glovebox. NMR spectra were recorded on a Bruker BioSpin GmbH spectrometer operating at 400.13 MHz (^1^H), 61.42 MHz (^2^H), 100.62 MHz (^13^C) and 56.2 MHz (^9^Be) or on an Agilent ProPulse spectrometer operating at 194.3 MHz (^7^Li). Solvents were dried by passage through a commercially available solvent purification system and stored under argon in ampules over 4 Å molecular sieves. C_6_D_6_ and C_7_D_8_ were purchased from Merck, and dried over potassium before distilling and storage over molecular sieves. {CH_2_SiMe_2_N(H)Dipp}_2_ and [{CH_2_SiMe_2_NDipp}_2_BeClLi] (**IV**^**Li**^) were synthesized according to literature procedures.^31,33^

### Synthesis of [{SiN^Dipp^}_2_BeHCs_2_N(H)Dipp]_2_ (**1**)

{SiN^Dipp^}Li(OEt_2_)_5_ (71 mg, 0.1 mmol) and BeCl_2_ (10 mg, 0.125 mmol) were introduced into a Young’s NMR tube and dissolved in protio-benzene (0.6 cm^3^), whereupon the sample was agitated and left to sit overnight before being filtered into a fresh NMR tube containing Cs metal. Upon contact with Cs an orange coloration and black precipitate was instantly observed. The reaction mixture was sonicated for 2 h and left to settle overnight before being filtered. Several colorless crystals of [{SiN^Dipp^}_2_Be–H–Cs_2_-N(H)Dipp]_2_ (**1**) were grown from the pale-orange solution at ambient temperature, though not enough for spectroscopic data. This compound was found to be unstable when exposed to vacuum. This product was also observed when reacting Cs with [{SiN^Dipp^ }_2_BeClLi]_2_ in protio-benzene.

### Synthesis of [Cs({SiN^Dipp^}BeD)]_2_ (**2**)

Cs metal was added to the top of a Young’s NMR tube containing [{SiN^Dipp^}_2_BeClLi]_2_ (**IV**^**Li**^ 17 mg, 0.030 mmol) in C_6_D_6_ (0.6 cm^3^). Upon melting the Cs (by hand), an orange coloration and black precipitate was instantly observed. The reaction mixture was sonicated for 2 h and left to settle overnight before being filtered. A small number of colorless crystals of [Cs({SiN^Dipp^}BeD)]_2_ (**2**) were grown from the pale-orange solution at ambient temperature. This compound was found to be unstable when exposed to vacuum. ^1^H NMR (C_6_D_6_): δ = 6.87 (d, *o-*Ar–H, ^*3*^*J*_*HH*_ = 7.25 Hz, 4H), 6.74 (t, *p-*Ar–H, ^*3*^*J*_*HH*_ = 7.46 Hz, 2H), 4.11 (sept, CH(CH_3_)_2_, ^*3*^*J*_*HH*_ = 6.52 Hz, 4H), 1.31 (d, CH(CH_3_)_2_, ^*3*^*J*_*HH*_ = 7.05 Hz, 12H), 1.19 (s, SiCH_2_, 4H), 0.93 (br d, CH(CH_3_)_2_, ^*3*^*J*_*HH*_ = 5.40 Hz, 12H), 0.2 (s, SiCH_3_, 12H). ^2^H NMR (C_6_D_6_): δ = 1.29 (s, Be-D). ^13^C{^1^H} NMR (C_6_D_6_): δ = 156.4 (*i-*C_6_H_3_), 147.3 (*m-*C_6_H_3_), 123.5 (*o-*C_6_H_3_), 121.1 (*p-*C_6_H_3_), 27.6 (CH(CH_3_)_2_), 24.8 (CH(CH_3_)_2_), 23.5 (CH(CH_3_)_2_), 14.8 (SiCH_2_), 1.0 (SiCH_3_). ^9^Be NMR (C_6_D_6_) δ = 11.8 (br s, ω_1/2_ = 353 Hz).

### Synthesis of [Cs({SiN^Dipp^}BeCD_2_C_6_D_5_)]_∞_ (**3**-*d*)

Cs metal was added to the top of a Young’s NMR tube containing [{SiN^Dipp^}_2_BeClLi]_2_ (**IV**^**Li**^, 20 mg, 0.035 mmol) in *d*_8_-toluene (0.6 cm^3^). Upon melting the Cs (by hand), an orange coloration and black precipitate was instantly observed. The reaction mixture was sonicated for 1 h and filtered. A small number of colorless crystals of [Cs({SiN^Dipp^}BeCD_2_C_6_D_5_)]_∞_ (**3-***d*) were grown from the pale-orange solution at ambient temperature. This compound was found to be unstable when exposed to vacuum. ^1^H NMR (C_7_D_8_): δ = 6.83 (d, *o-*Ar–H, ^*3*^*J*_*HH*_ = 7.60 Hz, 4H), 6.72 (t, *p-*Ar–H, ^*3*^*J*_*HH*_ = 7.60 Hz, 2H), 4.04 (sept, CH(CH_3_)_2_, ^*3*^*J*_*HH*_ = 6.84 Hz, 4H), 1.27 (d, CH(CH_3_)_2_, ^*3*^*J*_*HH*_ = 7.05 Hz, 12H), 1.09 (s, SiCH_2_, 4H), 0.88 (br d, CH(CH_3_)_2_, ^*3*^*J*_*HH*_ = 6.58 Hz, 12H), 0.1 (s, SiCH_3_, 12H). ^13^C{^1^H} NMR (C_6_D_8_): δ = 155.9 (*i-*C_6_H_3_), 146.7 (*m-*C_6_H_3_), 122.9 (*o-*C_6_H_3_), 120.6 (*p-*C_6_H_3_), 27.0 (CH(CH_3_)_2_), 24.2 (CH(CH_3_)_2_), 22.8 (CH(CH_3_)_2_), 14.0 (SiCH_2_), 0.4 (SiCH_3_). ^9^Be NMR (C_6_D_8_) δ = 11.6 (br s, ω_1/2_ = 306 Hz).

### Synthesis of [Cs({SiN^Dipp^}BeCH_2_C_6_H_5_)]_∞_ (**3**-*h*)

Cs metal was added to the top of a Young’s NMR tube containing [{SiN^Dipp^}_2_BeClLi]_2_ (**IV**^**Li**^, 17 mg, 0.030 mmol) in protio-toluene (0.6 cm^3^). Upon melting the Cs (by hand), an orange coloration and black precipitate was instantly observed. The reaction mixture was sonicated for 1 h and filtered. A small number of colorless crystals of [Cs({SiN^Dipp^}BeCH_2_C_6_H_5_)]_∞_ (**3-***h*) were grown from the pale-orange solution at ambient temperature. This compound was found to be unstable when exposed to vacuum. ^1^H NMR (C_6_D_6_): δ = Dipp aromatic protons swamped by solvent. 6.48 (t, benzyl *m-*Ar–H, ^*3*^*J*_*HH*_ = 7.59 Hz, 2H), 6.21 (t, benzyl *p-*Ar–H, ^*3*^*J*_*HH*_ = 7.48 Hz, 1H), 5.56 (d, benzyl *o-*Ar–H, ^*3*^*J*_*HH*_ = 7.79 Hz, 2H), 4.35 (sept, CH(CH_3_)_2_, ^*3*^*J*_*HH*_ = 6.84 Hz, 4H), 1.55 (d, CH(CH_3_)_2_, ^*3*^*J*_*HH*_ = 6.84 Hz, 12H), 1.40 (s, SiCH_2_, 4H), 1.25 (d, CH(CH_3_)_2_, ^*3*^*J*_*HH*_ = 6.58 Hz, 12H), 0.71 (s, benzyl CH_2_, 2H), 0.49 (s, SiCH_3_, 12H). ^13^C{^1^H} NMR (C_6_D_6_): δ = 156.2 (*i-*C_6_H_3_), 146.4 (*m-*C_6_H_3_), 127.4 (-benzyl), 127.3 (-benzyl), 123.0 (*o-*C_6_H_3_), 119.9 (*p-*C_6_H_3_), 117.4 (*p-*benzyl), 27.9 (CH(CH_3_)_2_), 25.7 (CH(CH_3_)_2_), 25.0 (CH(CH_3_)_2_), 23.9 (CH_2_–benzyl), 15.9 (SiCH_2_), 1.78 (SiCH_3_). ^9^Be NMR (C_6_D_6_) δ = 11.9 (br s, ω_1/2_ = 269 Hz).

## Results and Discussion

**Figure 2:**
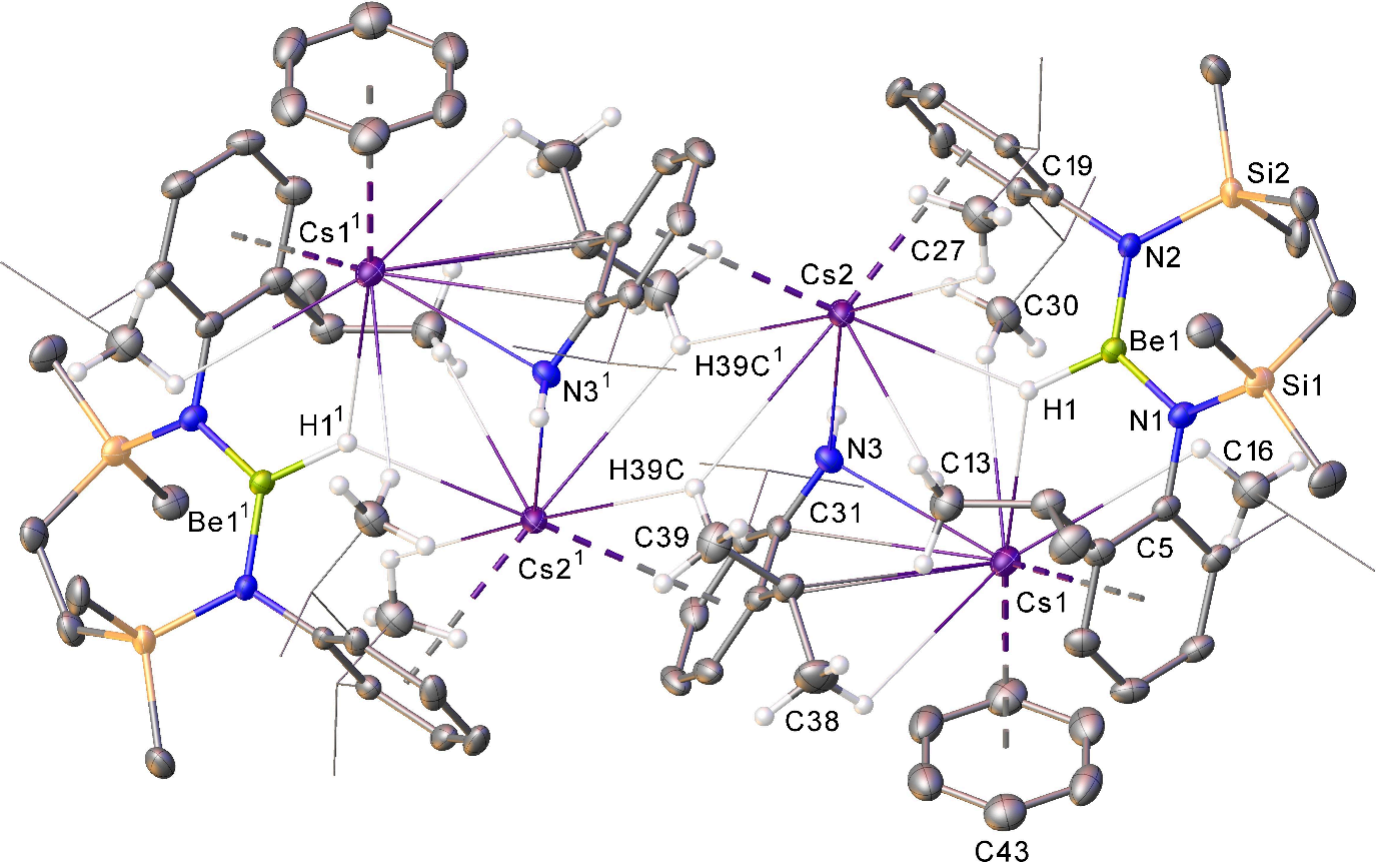
Displacement ellipsoid (30%) plot of compound **1**. For clarity, hydrogen atoms, except those attached to Be1 and N3 and those relevant to C–H···Cs close contacts, plus a molecule of occluded benzene solvent have been omitted. Additionally, the majority of isopropyl substituents are shown as wireframes, also for visual ease. Selected bond lengths (Å) and angles (deg): N1–Be1 1.623(5), N2–Be1 1.617(5), Cs1–N3 3.062(3), Cs1–C5 3.626(3), Cs1–C31 3.509(3), Cs2–N3 3.031(3), Cs2–C19 3.626(3), N2–Be1–N1 126.0(3). Symmetry operation to generate equivalent atoms ^1^1-*x*, 2-*y*, 1-*z.*

An initial reaction performed in protiobenzene at room temperature between **IV**^**Li**^ and an excess of Cs metal resulted in the generation of an orange solution and a black precipitate. Ultrasonication for 2 h, filtration, and crystallization at ambient temperature resulted in the isolation of several colorless single crystals of compound **1**, which, while insufficient in quantity to allow further spectroscopic characterization, were suitable for X-ray diffraction analysis. Although the resultant solid-state structure (Figure 2) identified compound **1** as a cesium hydridoberyllate comprising the previously suggested [{SiN^Dipp^}BeH]^−^ anion (Figure 1a), each molecule also incorporates a formal equivalent of the cesium anilide, [DippNHCs]. The gross structure of **1**, therefore, may be viewed as a centrosymmetric dimer in which each [{SiN^Dipp^}BeH]^−^ unit interacts with two cesium cations via a sequence of Be–H···Cs contacts and polyhapto arene···Cs interactions with the Dipp substituents of each {SiN^Dipp^} ligand. The more exposed Cs2 centers are further encapsulated through their η^6^ sequestration of a molecule of benzene solvent and a sequence of Cs1–N3/Cs1^1^–N3^1^ [3.062(3) Å] and Cs2–N3 [3.031(3) Å] close contacts to the anilide anions. Dimer propagation is also achieved via these latter anions, which act as bridging units through polyhapto engagement of Cs2/Cs2^1^, with the remaining unsaturation of the large Cs^+^ cations satisfied by C–H···Cs close contacts to the isopropyl groups of the various Dipp substituents. The beryllium {N_2_BeH} coordination environment of **1** is unambiguously three-coordinate and, although a number of molecular beryllium hydrides have now been structurally characterized,^34^ reports have been sporadic and the sole precedent with a similarly low coordination number was reported only very recently.^35^

**Figure 3:**
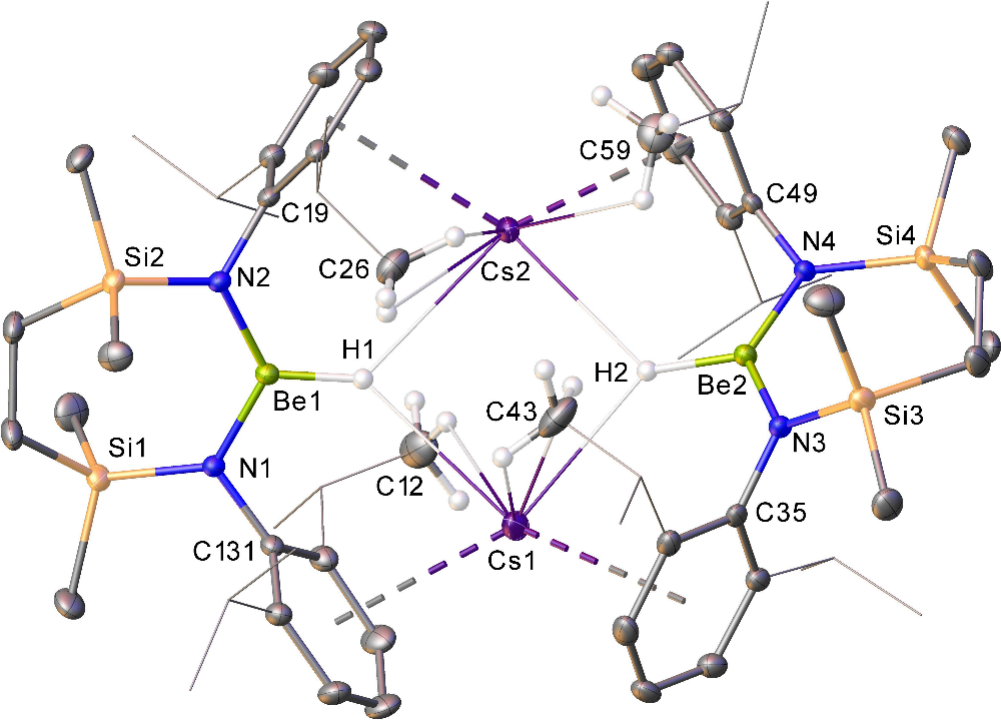
Displacement ellipsoid (30%) plot of compound **2**. For clarity, hydrogen atoms, except those attached to Be1 and N3 and those relevant to C–H···Cs close contacts, plus a molecule of occluded benzene solvent, have been omitted. Additionally, the majority of isopropyl substituents are shown as wireframes, also for visual ease. Selected bond lengths (Å) and angles (deg): N1–Be1 1.623(5), N2–Be1 1.617(5), Cs1–N3 3.062(3), Cs1–C5 3.626(3), Cs1–C31 3.509(3), Cs2–N3 3.031(3), Cs2–C19 3.626(3), and N2–Be1–N1 126.0(3). Symmetry operation to generate equivalent atoms ^1^1-*x*, 2-*y*, 1-*z.*

**Scheme 1:**
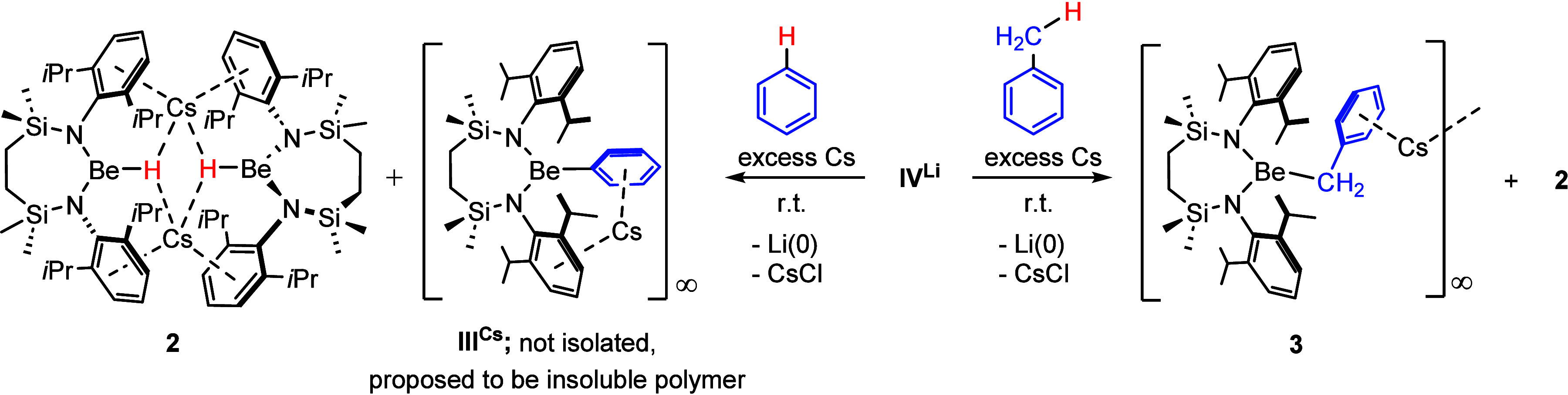
Proposed Course of Reaction of Compound **IV**^**Li**^ with Cesium Metal Leading to the Isolation of Compounds **2** and **3**

Although ^1^H NMR analysis of the supernatant collected after the isolation of the crystalline sample of **1** evidenced a complex reaction that frustrated confident interpretation (Figure S1), its structure confirms that reduction of the lithium cation of **IV**^**Li**^ can ensue alongside beryllium hydride formation. The observation of the free anilide anion within its structure, however, indicated that competitive degradation of the {SiN^Dipp^} ligand structure had also occurred, despite the mild room temperature reaction conditions. In an attempt to obtain more meaningful diagnostic analysis of these processes by NMR spectroscopy, therefore, a further reaction between **1** and Cs metal was undertaken in C_6_D_6_. Although a similar outcome was anticipated, the resultant ^1^H NMR spectrum was consistent with significantly enhanced kinetic discrimination and the generation of a predominant new product (**2**, Scheme 1). Notably, the similarity of this spectrum to the analogous data provided by all five of the previously described chloroberyllate species, **IV**^**M**^ (M = Li, Na, K, Rb, Cs), was indicative of a comparably symmetrical structure. Similarly, the single resonant frequency presented by the corresponding ^9^Be NMR experiment (δ = 11.8 ppm), and the breadth of this signal (ω_1/2_ = 353 Hz), were consistent with the maintenance of a similar 3-coordinate and significantly asymmetric beryllium binding environment.^36^ The origin of these observations was again resolved through the isolation and X-ray diffraction analysis of single crystals of compound **2** (Scheme 1, Figure 3). This experiment further confirmed the validity of our previous assumption of hydridoberyllate formation under such reductive reaction conditions. Although its gross structure demonstrates a close comparison to those of the similarly dimeric but chloride-bridged species, **IV**^**M**^, the 7-membered chelate dimer halves of both independent molecules within the asymmetric unit display a notably more twisted disposition of the {SiN^Dipp^}-chelated Be-containing units, presumably a consequence of the lower steric demands and directionality of binding to the hydride substituent. The relevant N–Be–N least-squares planes consequently subtend dihedral angles of 69.87 and 68.11° for the Be1/Be2- and Be3/Be4-containing molecules, respectively.

Additional solution analysis of the isolated crystals of compound **2** by ^2^H NMR spectroscopy in C_6_D_6_ identified a singlet resonance at δ 1.29 ppm, which we assign to the beryllium-bound deuteride signal arising from activation of the C_6_D_6_ solvent. The *in situ* solution analysis of the reaction leading to the isolation of compound **2**, however, provided no evidence for the simultaneous generation of a cesium species (**III**^**Cs**^) analogous to the previously reported lithium and sodium phenylberyllate derivatives, **III**^**Li**^ and **III**^**Na**^ (Figure 1a).^31^ Notably, the solid-state structure of **III**^**Na**^ was observed to be a 1-dimensional polymer propagated by intra- and intermolecular polyhapto engagement of the Na^+^ cations with the beryllium-bound phenyl substituent and the N-Dipp π-system of an adjacent SiN^Dipp^ spectator ligand. On this occasion, therefore, we suggest that potential for similar polymerization is exacerbated by the significantly larger radius of the cesium cation of the supposed [Cs({SiN^Dipp^}BePh)]_∞_ (**III**^**Cs**^),^37^ such that this compound most likely comprises a significant component of the insoluble material formed during the reaction.

In a further attempt to provide corroborative evidence for the simultaneous generation of both hydrido- and organoberyllium products of arene solvent activation, the reaction of **IV**^**Li**^ and Cs metal was repeated in *d*_8_-toluene. This process resulted in the familiar observation of a black precipitate and orange solution, analysis of which by ^1^H NMR spectroscopy presented signals strongly reminiscent of the initial data provided by compound **2**. A singlet resonance that could also be discriminated at δ 1.35 ppm in the corresponding ^2^H NMR spectrum was tentatively assigned as the Be-D signal of a deuteride species analogous to that assigned for **2**, albeit with a slight discrepancy in chemical shift induced by the differing deuterated solvents.

**Figure 4:**
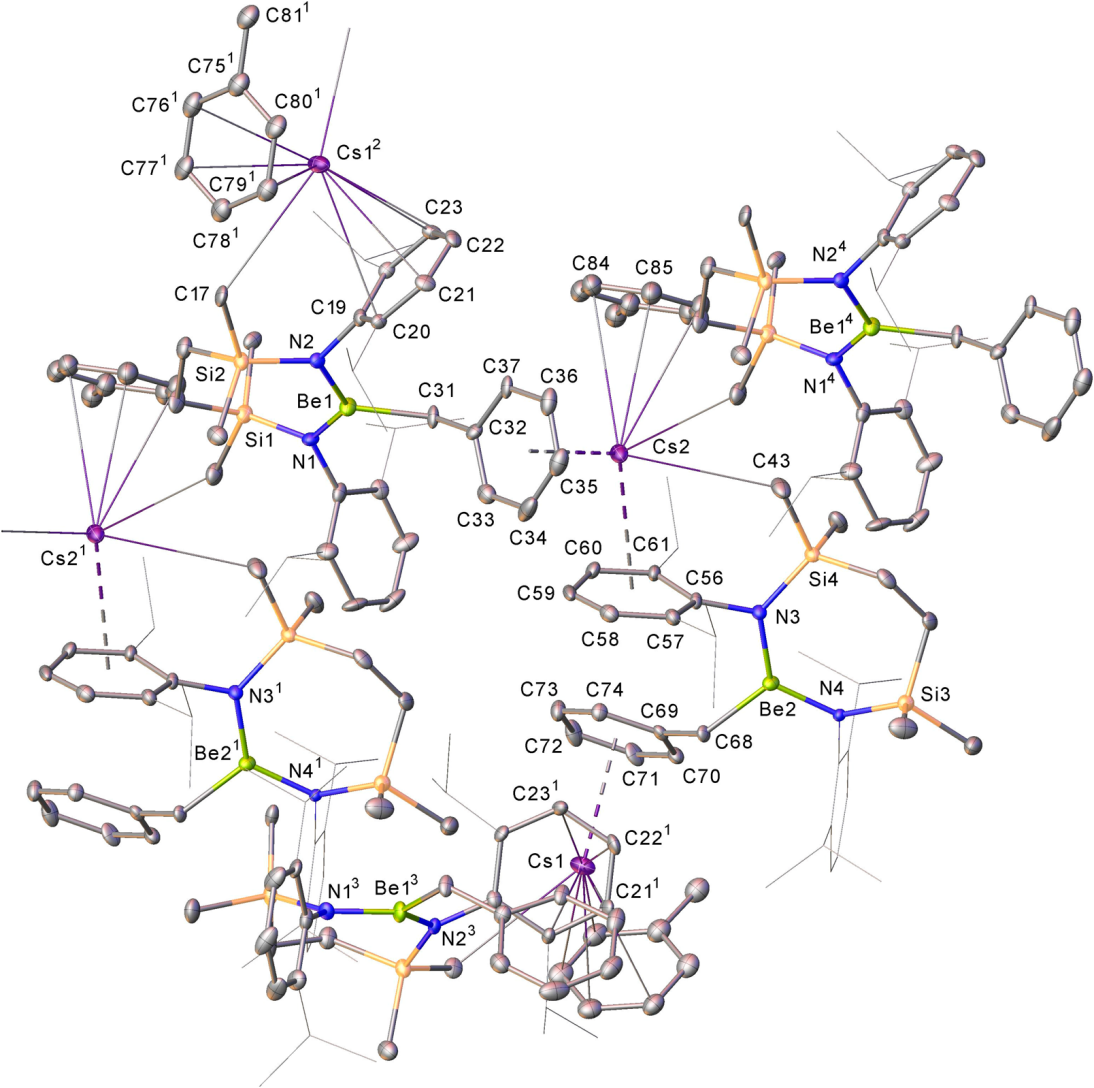
Displacement ellipsoid (30%) plot of a section of the 1-dimensional polymeric array of compound **3**. Hydrogen atoms and disordered atoms have been omitted, and *iso*-propyl substituents are shown as wire frames for clarity. Selected bond lengths (Å) and angles (deg): N1–Be1 1.60(2), N2–Be1 1.66(2), N3–Be2 1.65(2), N4–Be2 1.66(2), N1–Be1–N2 125.0(15), N3–Be2–N4 124.9(14). Symmetry operations to generate equivalent atoms: ^1^*x*, – 1 + *y*, *z*; ^2^1/2 – *x*, *y*, – 1/2 + *z*; ^3^1/2 – *x*, *y*, – 1/2 + *z*; ^4^*x*, 1 + *y*, *z*.

Although filtration and crystallization of the reaction solution did not allow definitive solid-state authentication of the formation of **2**, the resultant crystals were identified by X-ray diffraction analysis as a further product of beryllium-centered activation of the arene solvent (**3-***d*, Figure 4). In this case, C–D bond cleavage occurs at the thermodynamically preferred methyl substituent to provide the perdeuterobenzylberyllate species, [Cs({SiN^Dipp^}BeCD_2_C_6_D_5_)]_∞_. The resultant solid-state structure of compound **3-***d* is, thus, reminiscent of that previously observed for **III**^**Na**^ in comprising a 1-dimensional polymeric array. In the current case, the larger cesium cations of the molecular [Cs({SiN^Dipp^}BeCD_2_C_6_D_5_)] units are coordinated not only by the pi-system of each beryllium-bound benzylic substituent but also by a molecule of toluene solvent. The cesium coordination sphere is completed by further intermolecular Cs···arene interactions with a Dipp substituent of each adjacent [Cs({SiN^Dipp^}BeCD_2_C_6_D_5_)] moiety to generate a polymeric helix, the individual strands of which pack as supramolecular sheets stacked along the *b* axis defined by the orthorhombic unit cell (Figure S20).

Mindful of the kinetic discrimination provided by the otherwise identical reactions performed in perdeutero- and protio-benzene, compound **1** was also reacted with cesium metal in protio-toluene. Although the course of the reaction in this case appeared to proceed in a fashion analogous to that observed in the deuterated medium, analysis of the resultant crystals of compound **3-***h* in C_6_D_6_ enabled assignment of the respective benzylic methylene and aromatic resonances by ^1^H NMR spectroscopy (Figure S15).

## Conclusions

In conclusion, we have observed that a dimeric lithium chloroberyllate reacts at room temperature with cesium metal to provide cesium hydrido- and organoberyllate products resulting from C–H activation of the benzene or toluene solvent. These observations lend further credence to our earlier hypothesis that such arene activation is best rationalized as a result of highly reactive and short-lived Be(I) radical anion intermediates. We are thus continuing to study this and related reactivity with a view toward achieving the more direct observation or isolation of relevant lower oxidation state group 2 species.

## Supporting Information Available

General procedures, NMR spectra, and crystallographic data (PDF)
