# Supplementary material for: Cesium Reduction of a Lithium Diamidochloroberyllate
Source: Organometallics. 2024 Jan 24;43(3):432–7. doi: 10.1021/acs.organomet.3c00519 (PMC10865438; doi:10.1021/acs.organomet.3c00519)
Supplement: Supplementary file 1 — om3c00519_si_001.pdf [file om3c00519_si_001.pdf]

Supplementary Information for

Cesium Reduction of a Lithium Diamidochloroberyllate

Kyle G. Pearce, Michael S. Hill* and Mary F. Mahon*

Department of Chemistry, University of Bath, Claverton Down, Bath, BA2 7AY, United Kingdom

Email: msh27@bath.ac.uk; m.f.mahon@bath.ac.uk

NMR Spectra

Figures S1-S19: NMR Spectra **S2-S12**

Crystallographic Details **S13**

Table S1: Crystal data and structure refinement for compounds **1**, **2** and **3**-***d***. **S15**

References **S16**

NMR Spectra

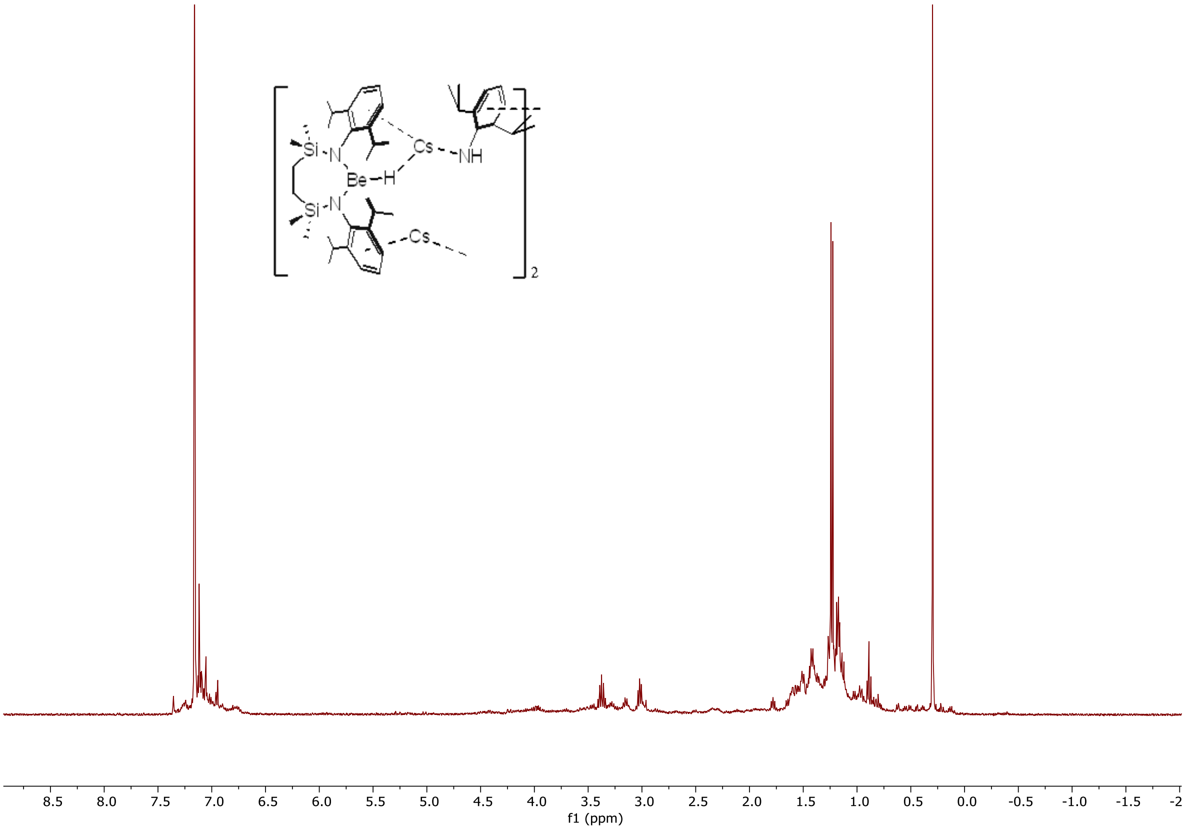

Figure S1. *^{1}H NMR Spectrum (C_6D_8, 298 K, 400.13 MHz) for* ***$[\{SiN^{Dipp}\}_2Be\text{-}H\text{-}Cs_2\text{-}N(H)Dipp]_2$ (1)*.**

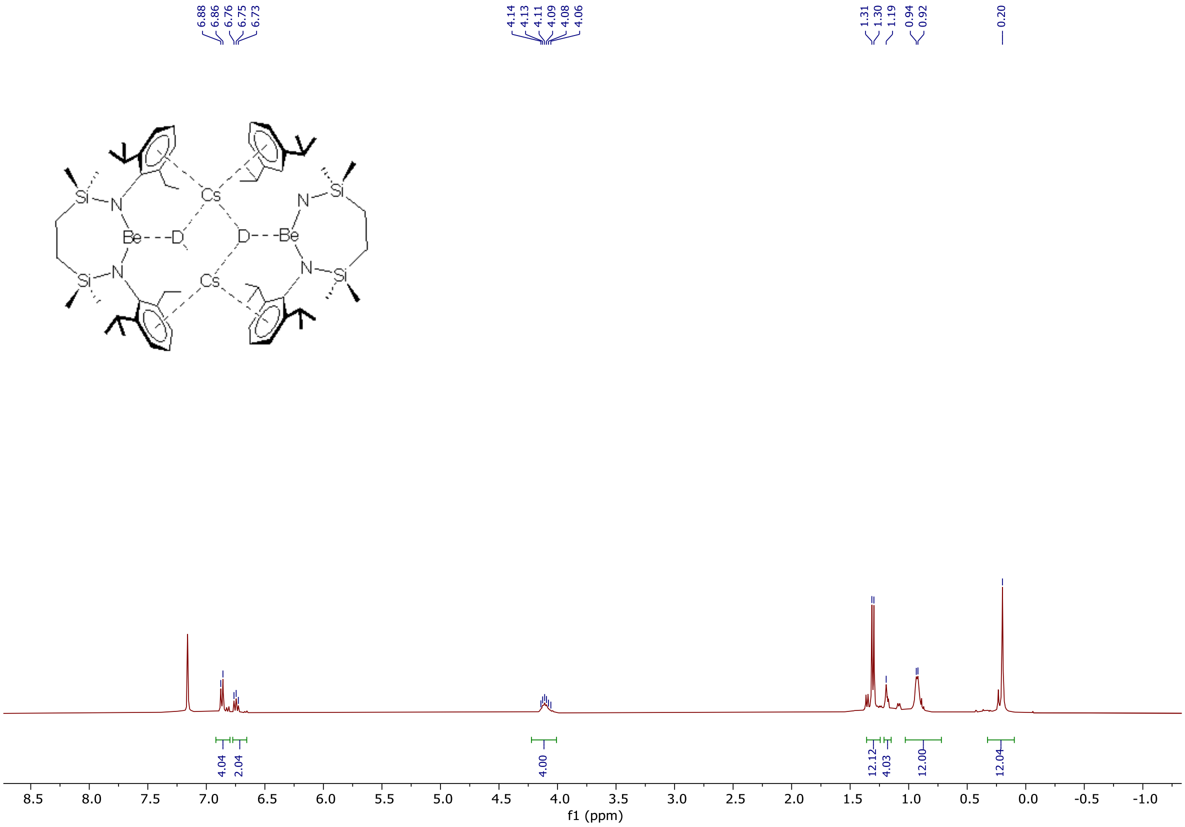

Figure S2. *^{1}H NMR Spectrum (C_6D_6, 298 K, 400.13 MHz) for* ***[Cs({SiN^{Dipp}}BeD)]$_2$ (2)***.

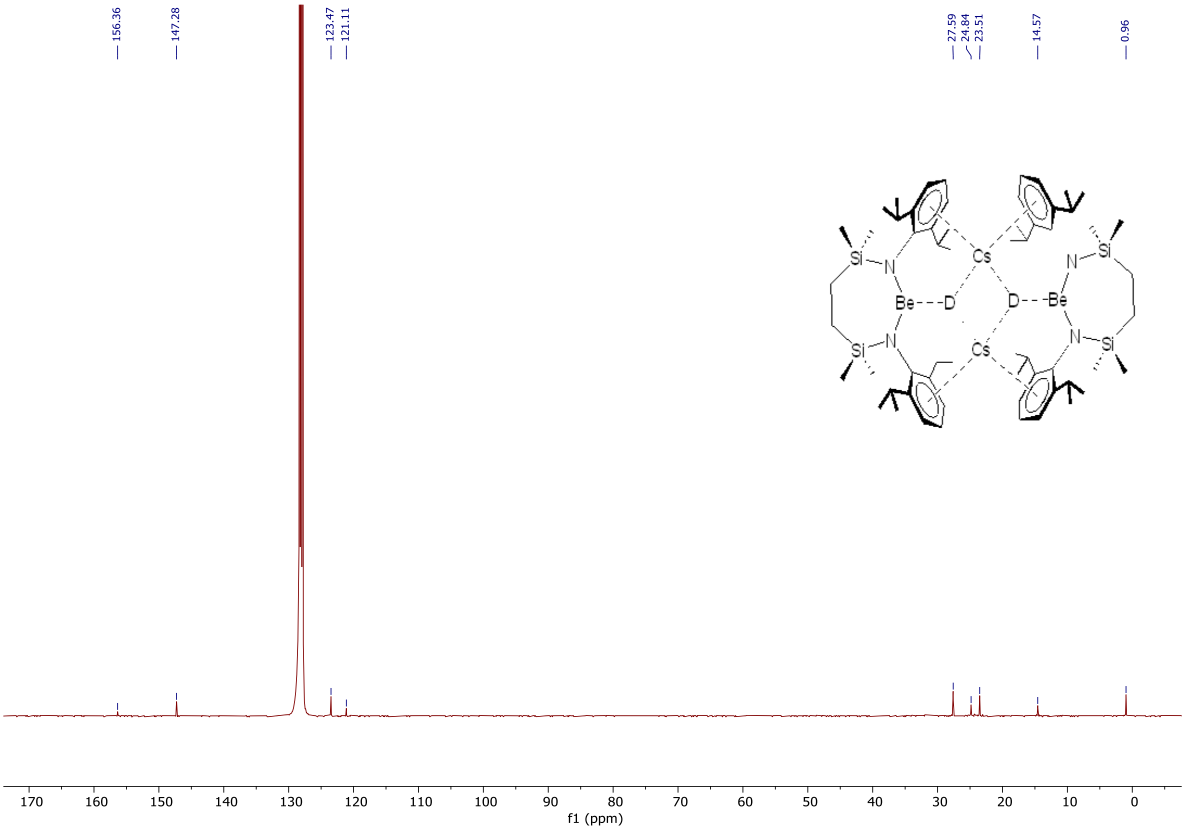

Figure S3. *^{13}C{^{1}H} NMR Spectrum (C_6D_6, 298 K, 100.62 MHz) for* ***[Cs({SiN^{Dipp}}BeD)]$_2$ (2)***.

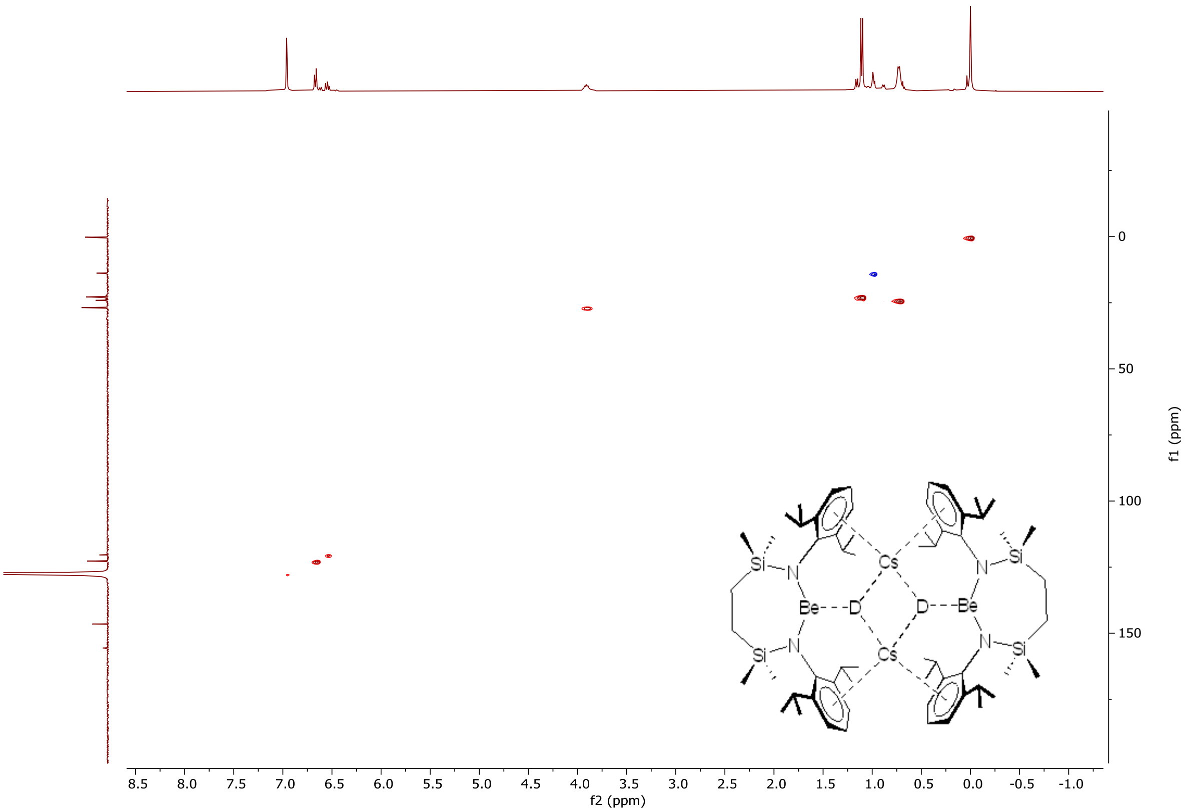

Figure S4. *^{1}H-^{13}C HSQC trace (C_6D_6, 298 K, 400.13, 100.62 MHz) for* ***[Cs({SiN^{Dipp}}BeD)]$_2$ (2)*****.**

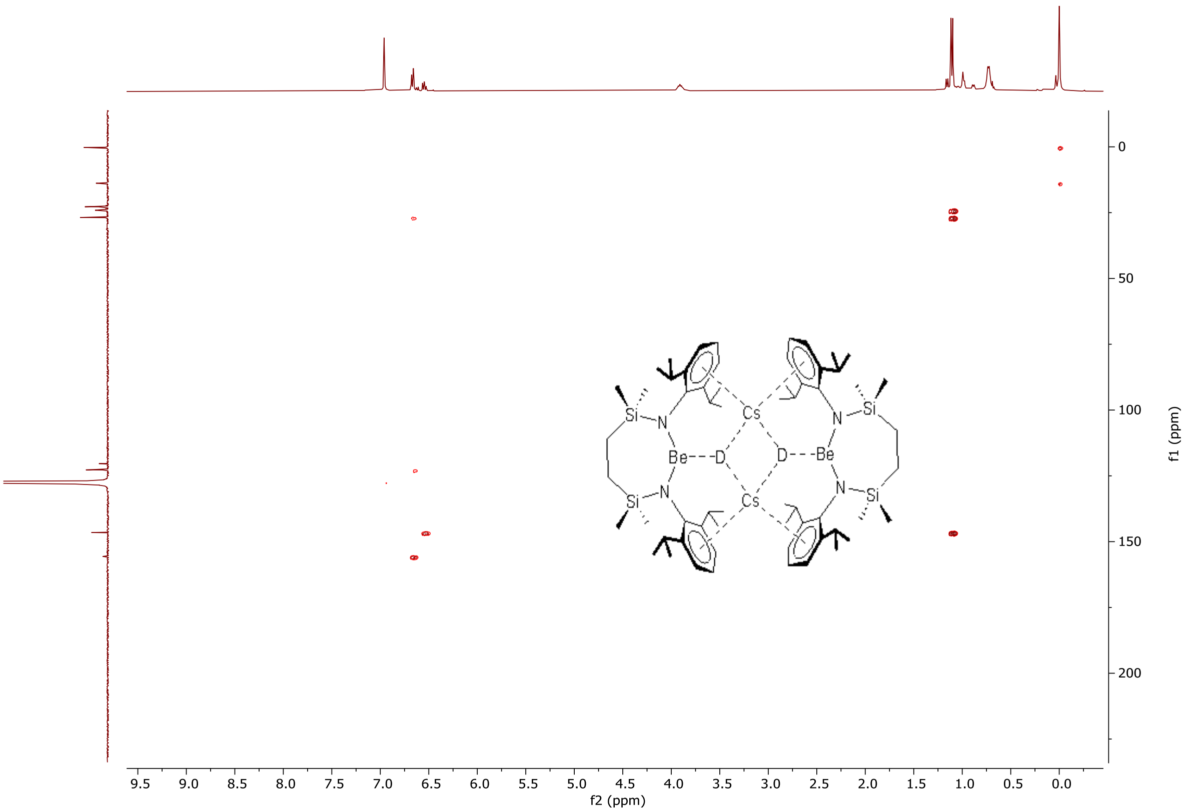

Figure S5. *^{1}H-^{13}C HMBC trace (C_6D_6, 298 K, 400.13, 100.62 MHz) for* ***[Cs({SiN^{Dipp}}BeD)]$_2$ (2)*****.**

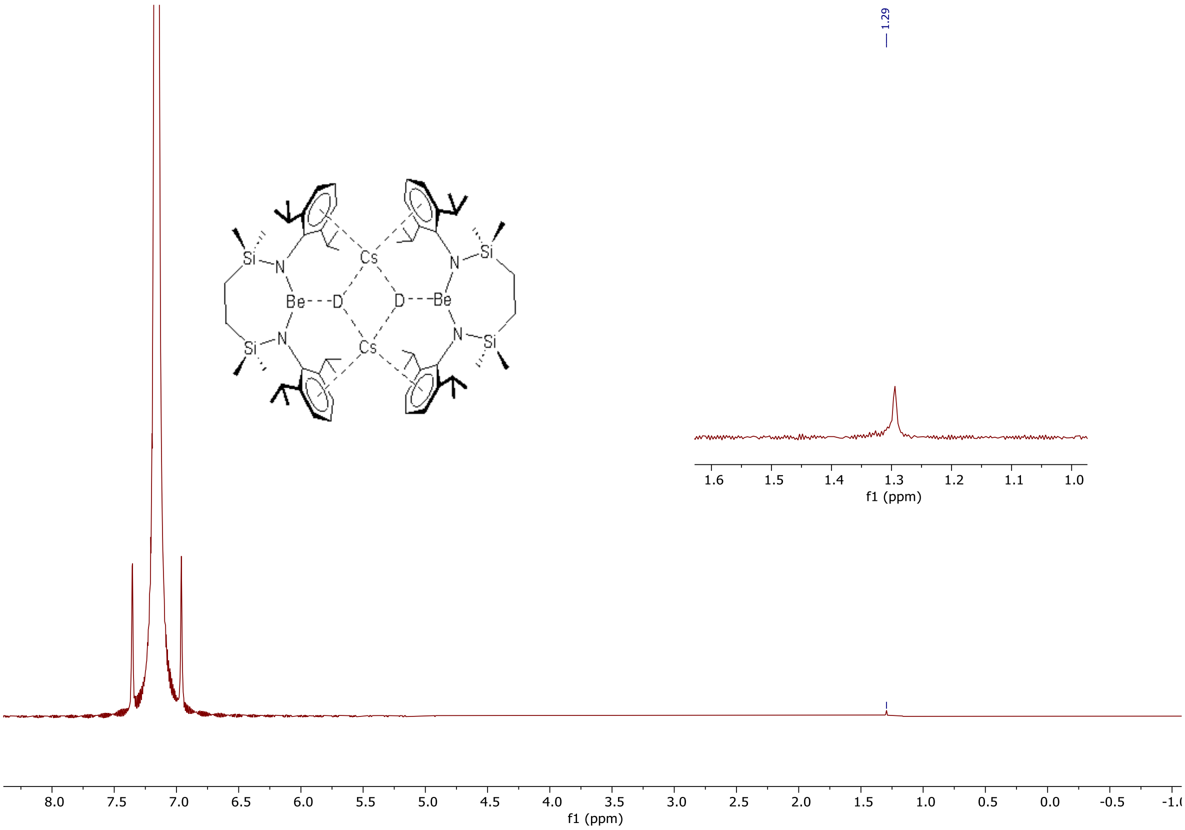

Figure S6. *²H NMR Spectrum (C_6D_6, 298 K, 61.42 MHz) for* ***[Cs({SiN^{Dipp}}BeD)]$_2$ (2)*****.**

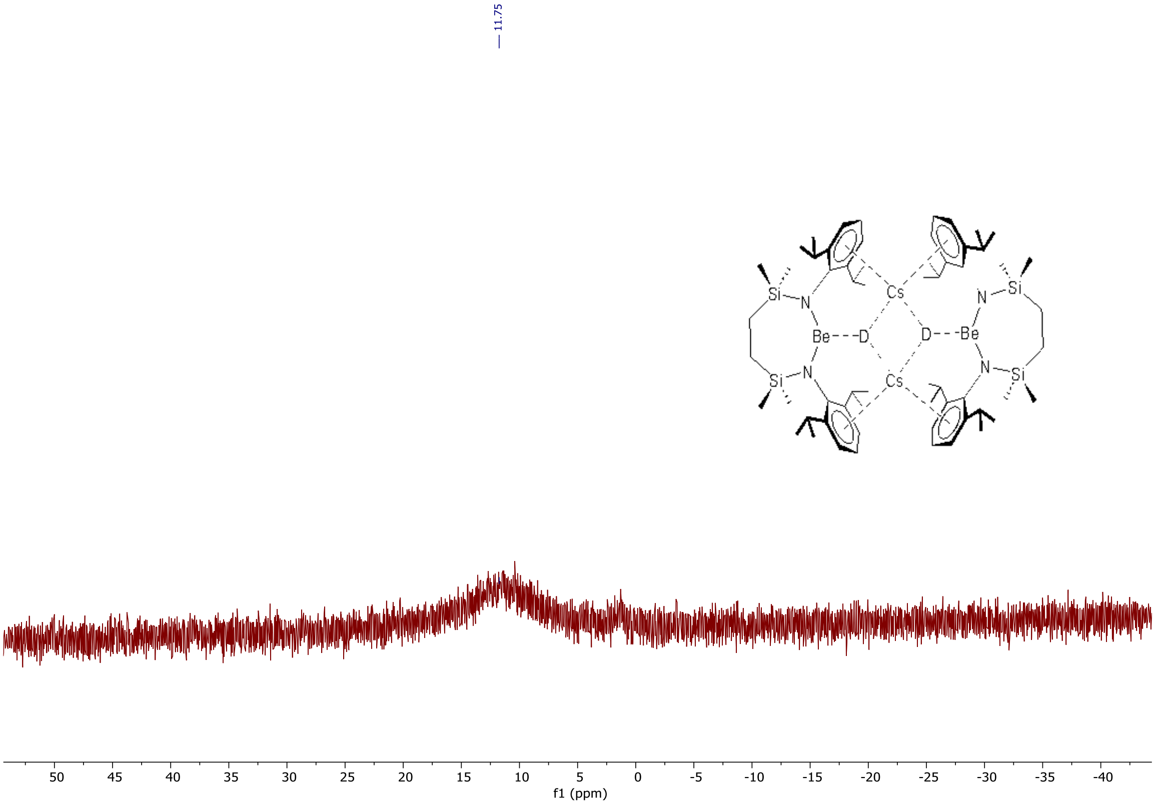

Figure S7. *⁹Be NMR Spectrum (C_6D_6, 298 K, 56.2 MHz) for* ***[Cs({SiN^{Dipp}}BeD)]$_2$ (2)*****.**

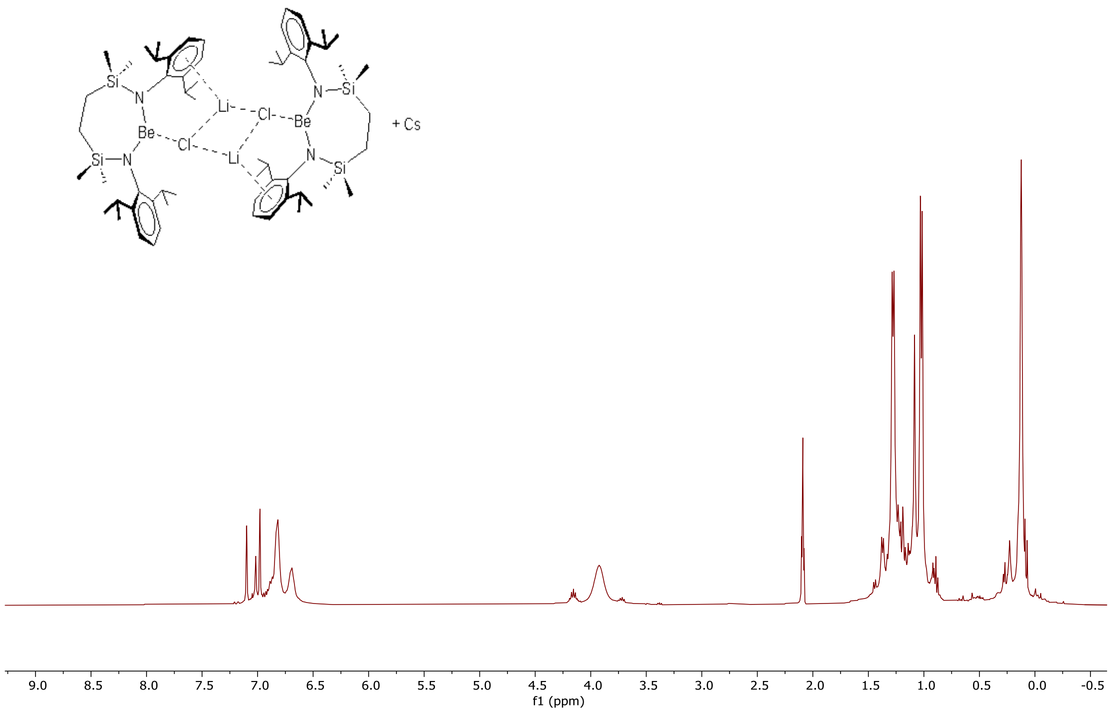

Figure S8. *^{1}H NMR Spectrum (C_6D_8, 298 K, 400.13 MHz) of the initial reaction mixture of [{SiN^{Dipp}}$_2$BeClLi]$_2$ (**IV^{Li}**) and Cs in d_8-toluene.*

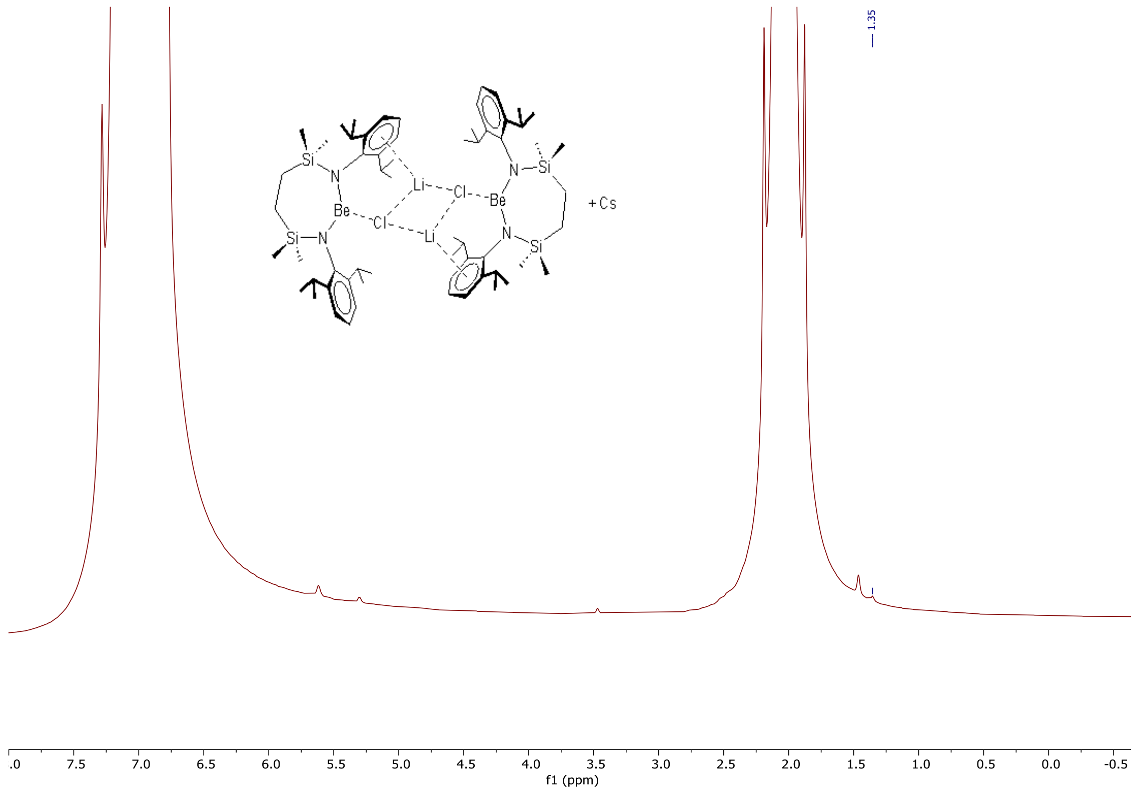

Figure S9. *^{2}H NMR Spectrum (C_6D_8, 298 K, 61.42 MHz) of the reaction mixture of [{SiN^{Dipp}}$_2$BeClLi]$_2$ (**IV^{Li}**) and Cs in d$_8$-toluene after filtration.*

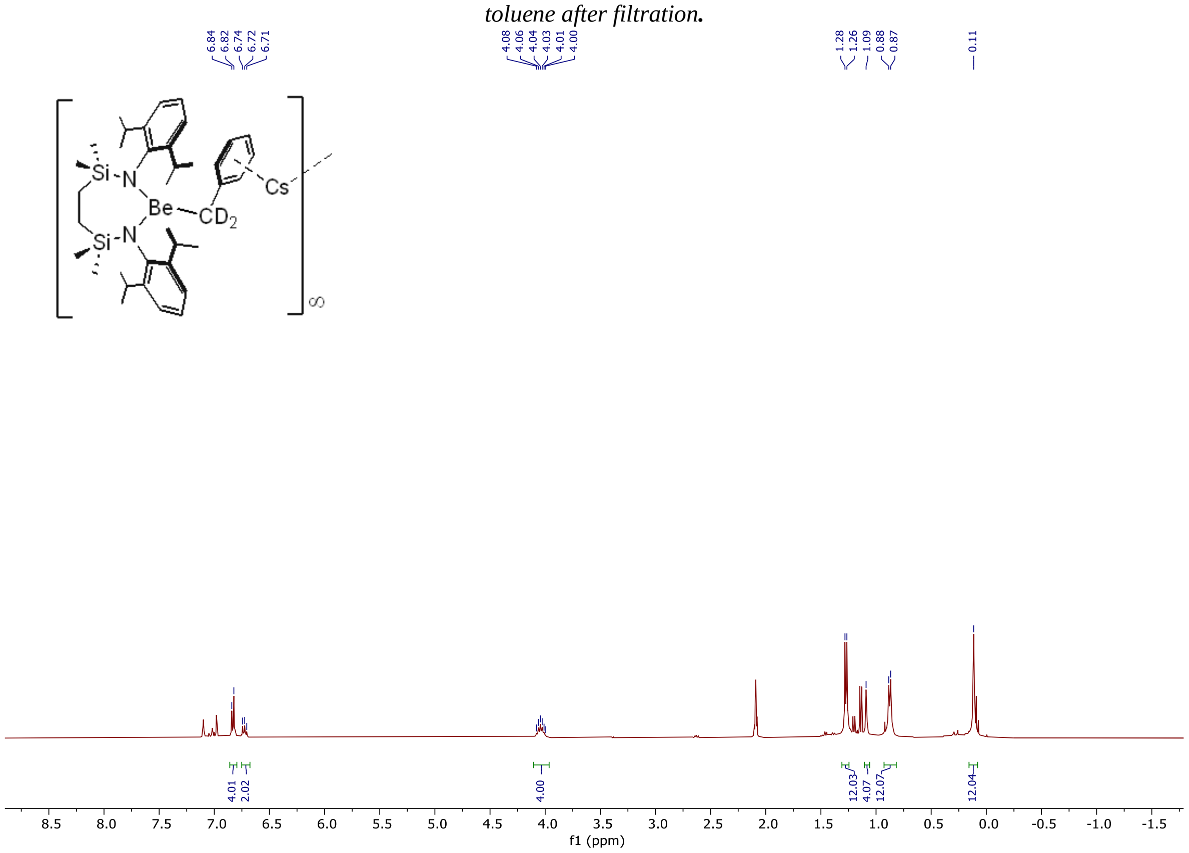

Figure S10. *^{1}H NMR Spectrum (C_6D_8, 298 K, 400.13 MHz) for* **[Cs({SiN^{Dipp}}BeCD$_2$C$_6$D$_5$)] (3-d)**.

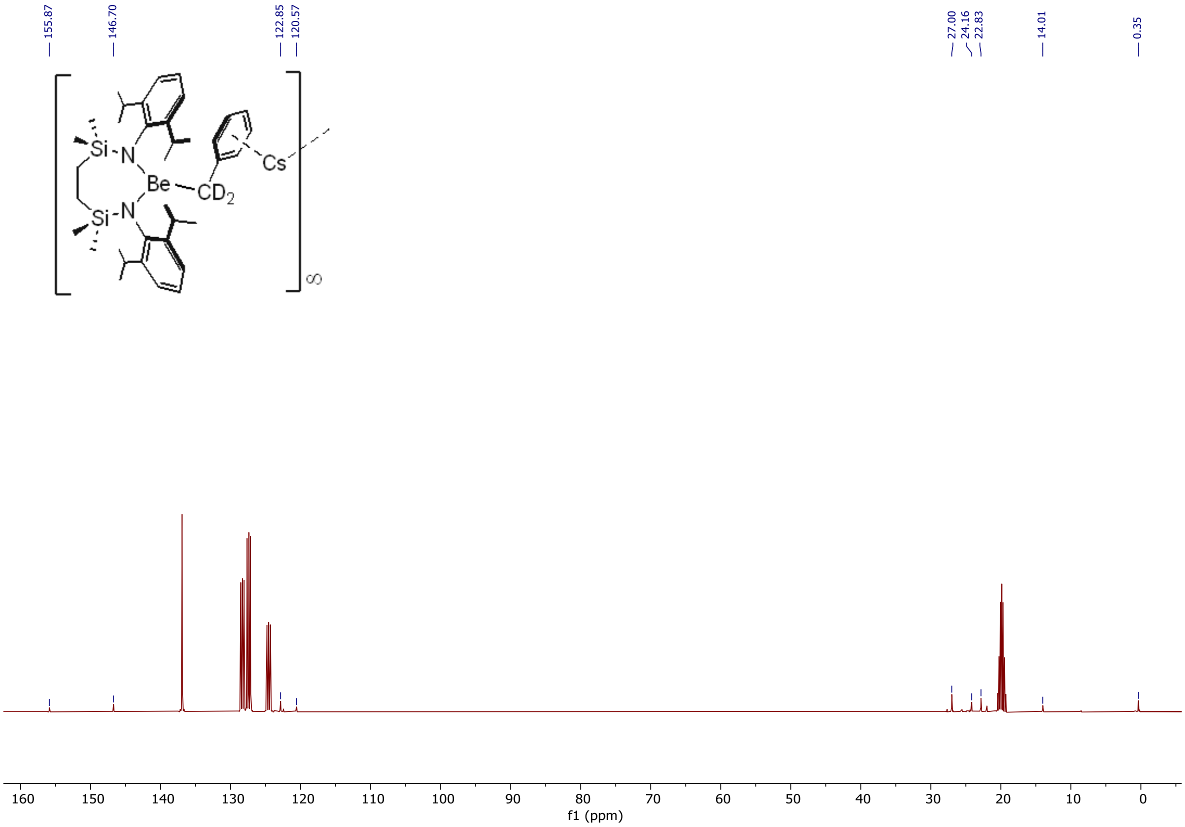

***Figure S11**. $^{13}C\{^{1}H\}$ NMR Spectrum (C_6D_8, 298 K, 100.62 MHz) for* ***[Cs({SiN^{Dipp}}BeCD$_2$C$_6$D$_5$)]$_\infty$ (3-d)**.*

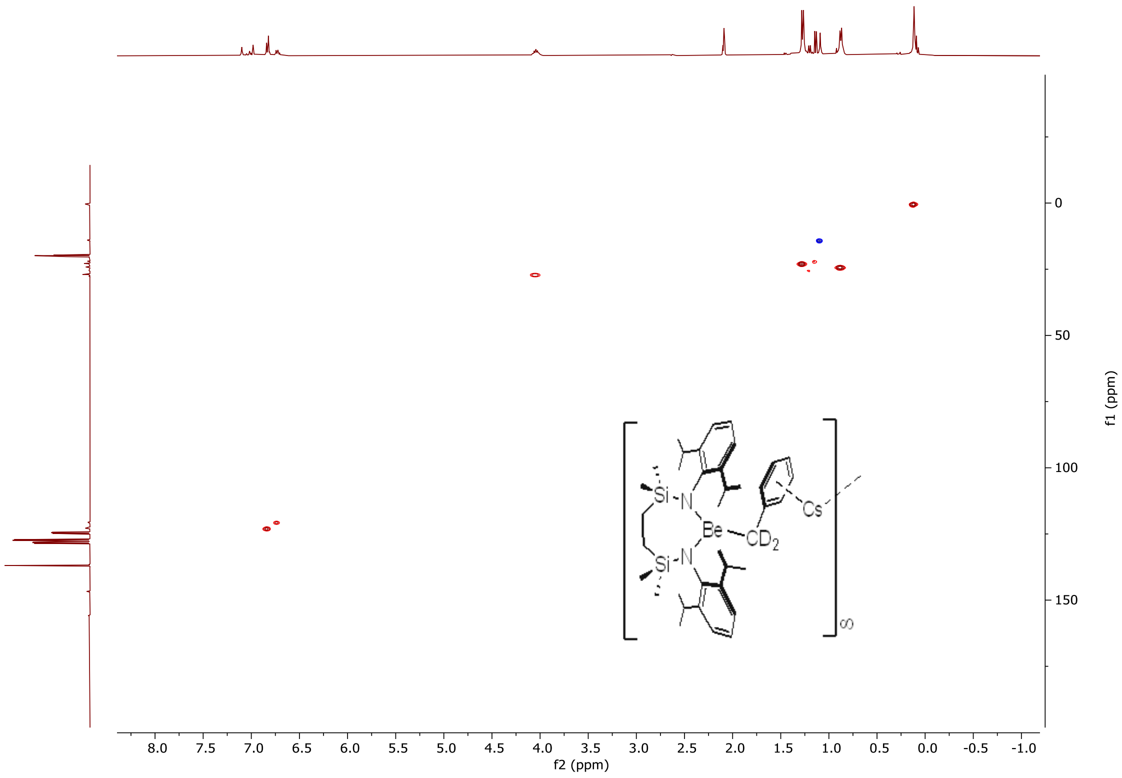

***Figure S12.** ^{1}H-^{13}C HSQC trace (C_6D_8, 298 K, 400.13, 100.62 MHz) for* ***[Cs({SiN^{Dipp}}BeCD$_2$C$_6$D$_5$)]$_\infty$ (3-d)**.*

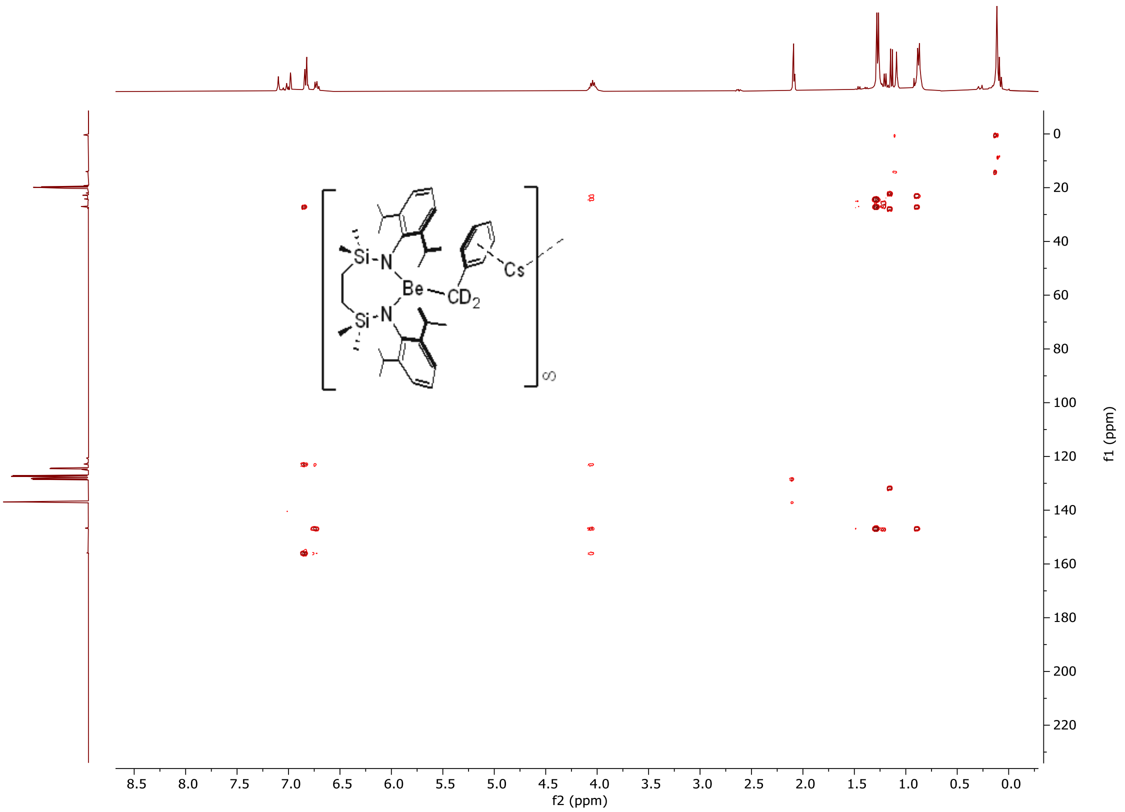

Figure S13. ^{1}H-^{13}C HMBC trace (C_6D_8, 298 K, 400.13, 100.62 MHz) for **[Cs({SiN^{Dipp}}BeCD$_2$C$_6$D$_5$)]$_\infty$ (3-d).**

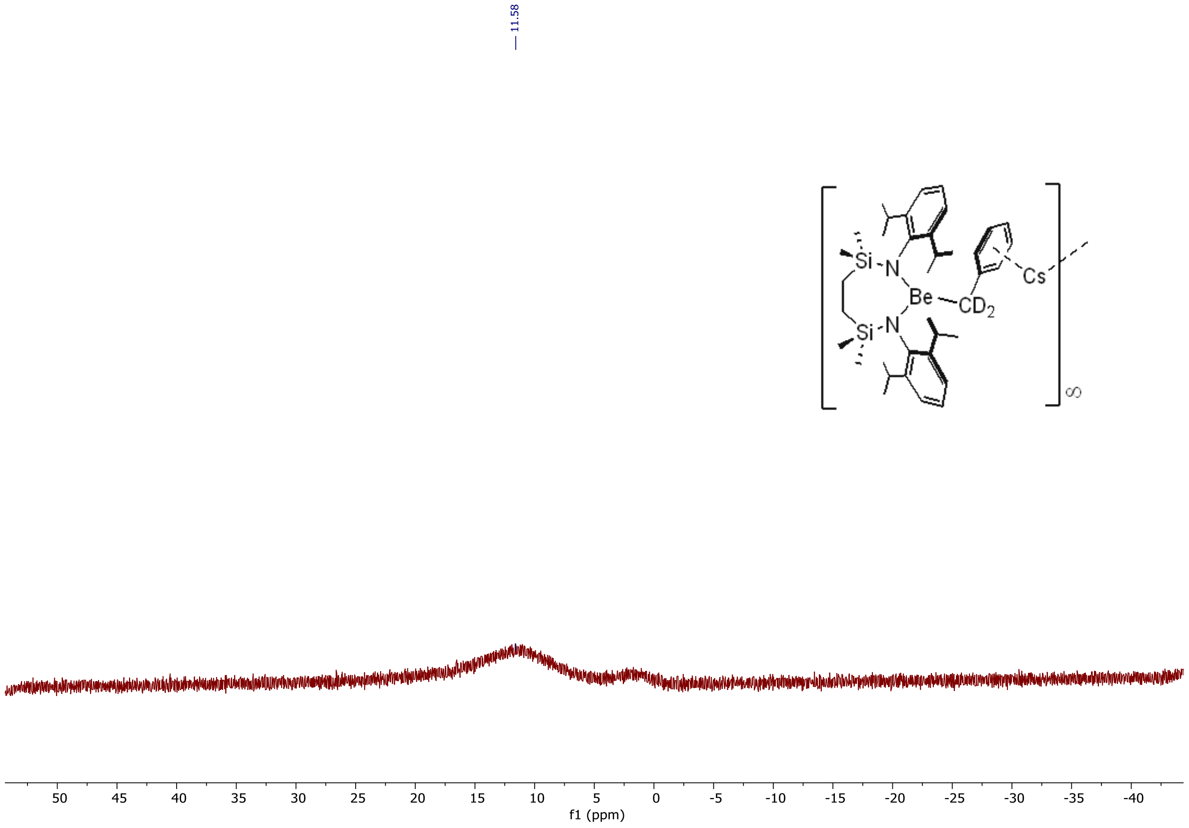

Figure S14. [9]Be NMR Spectrum (C_6D_8, 298 K, 56.2 MHz) for **[Cs({SiN^{Dipp}}BeCD$_2$C$_6$D$_5$)]$_\infty$ (3-d).**

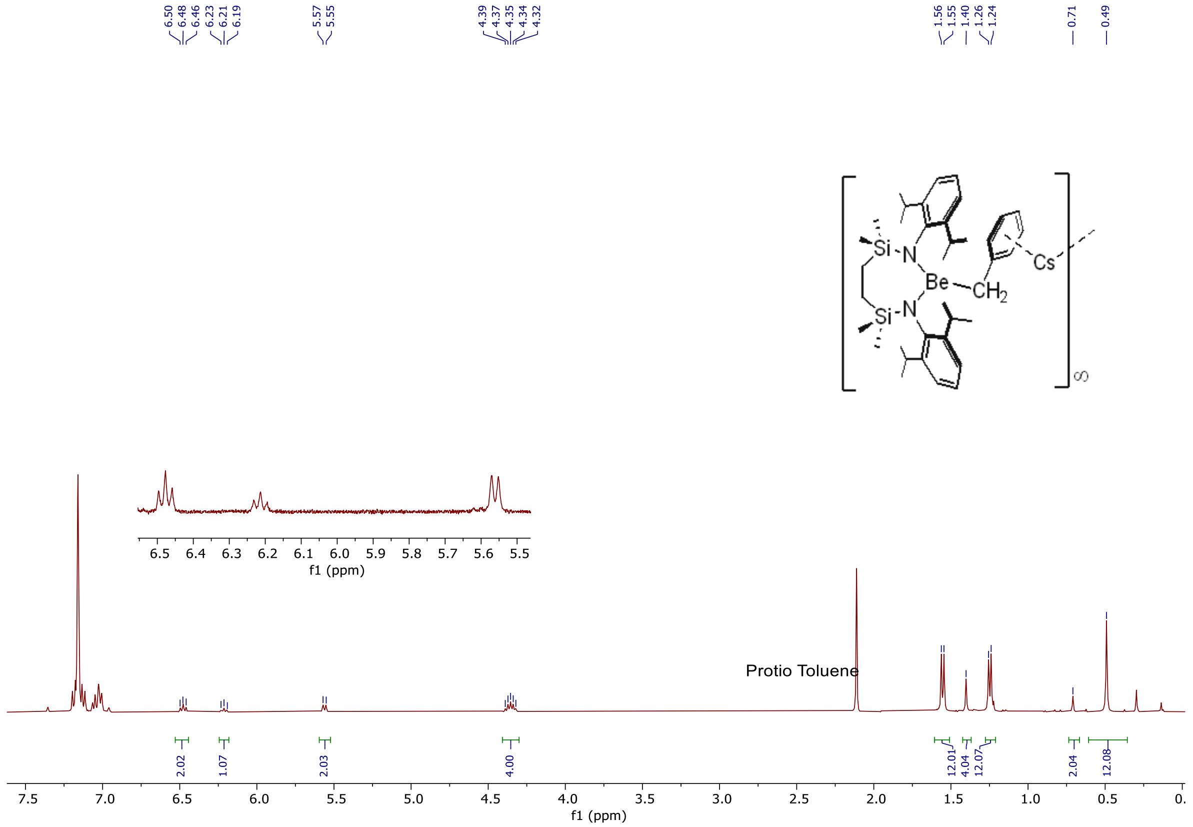

Figure S15. *^{1}H NMR Spectrum (C_6D_6, 298 K, 400.13 MHz) for* ***[Cs({SiN^{Dipp}}BeCH$_2$C$_6$H$_5$)]$_\infty$ (3-h)*****.**

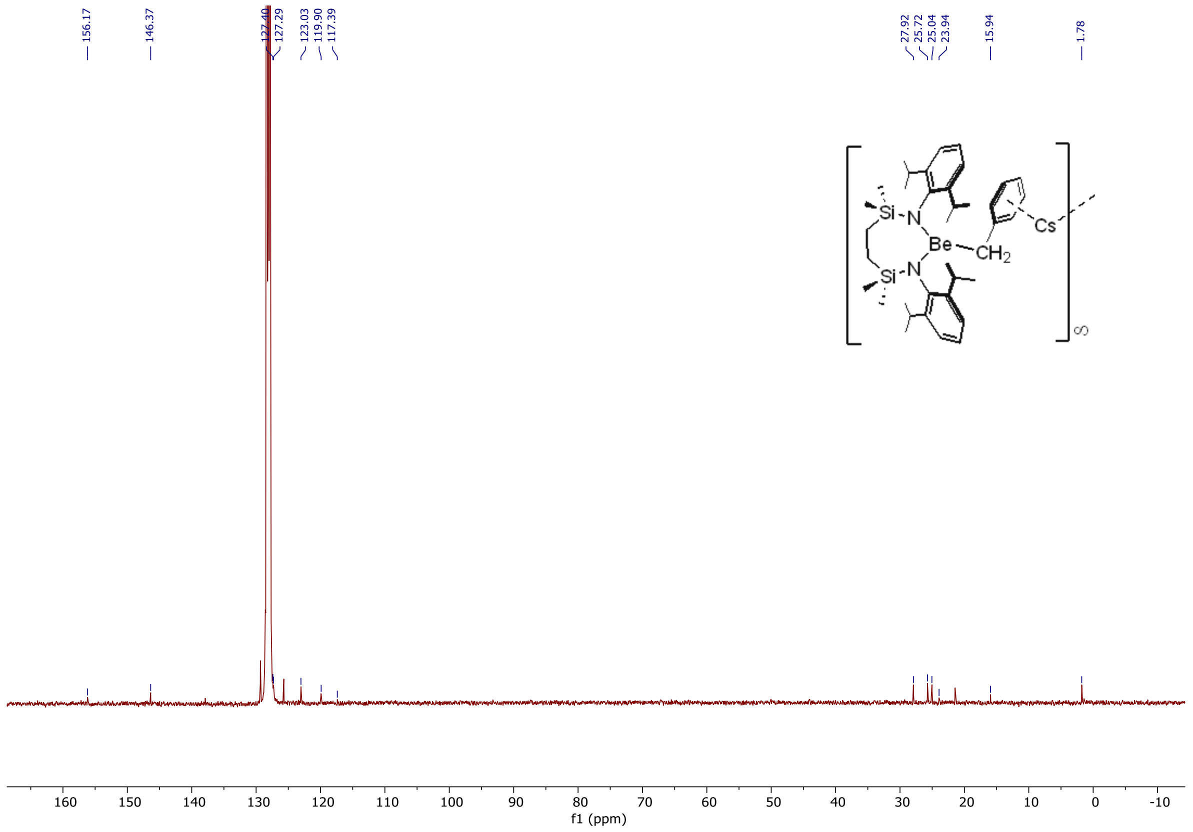

Figure S16. *^{13}C{^{1}H} NMR Spectrum (C_6D_6, 298 K, 100.62 MHz) for* ***[Cs({SiN^{Dipp}}BeCH$_2$C$_6$H$_5$)]$_\infty$ (3-h)*****.**

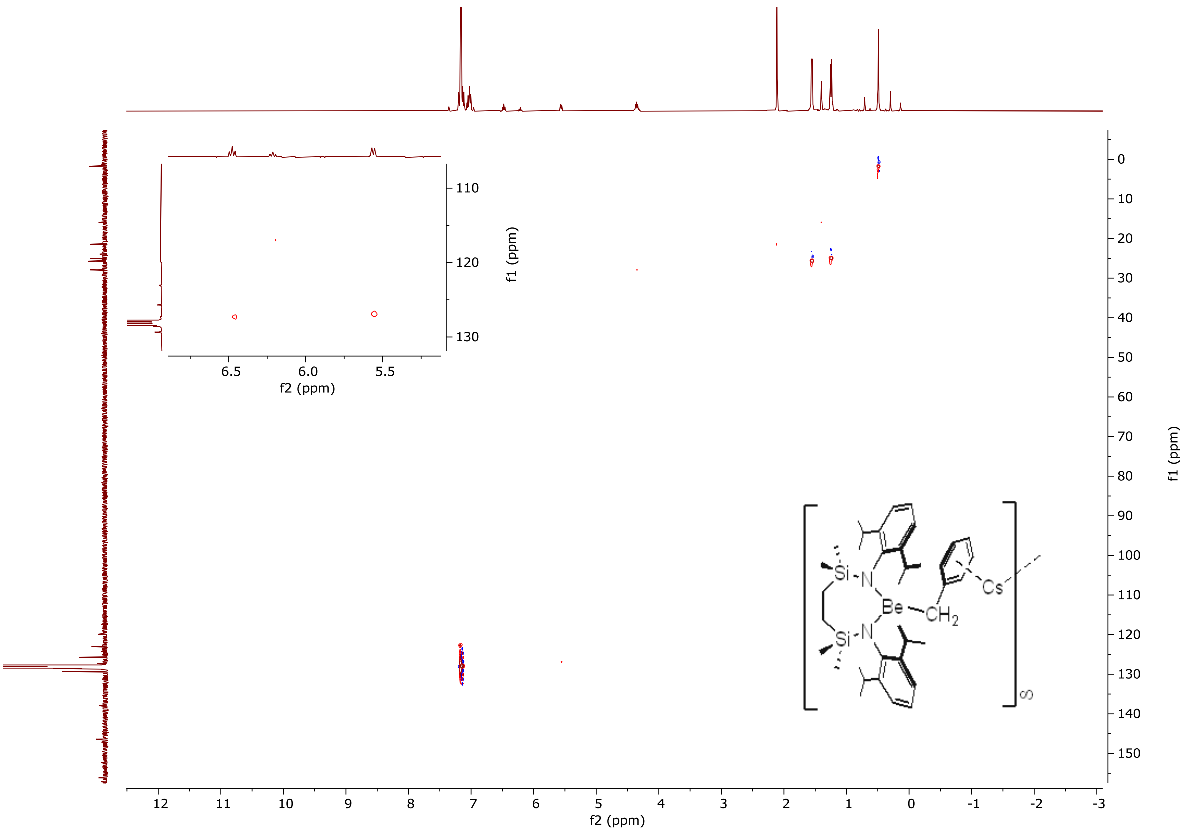

Figure S17. *^{1}H-^{13}C HSQC trace (C_6D_6, 298 K, 400.13, 100.62 MHz) for* ***[Cs({SiN^{Dipp}}BeCH$_2$C$_6$H$_5$)]$_\infty$ (3-h).***

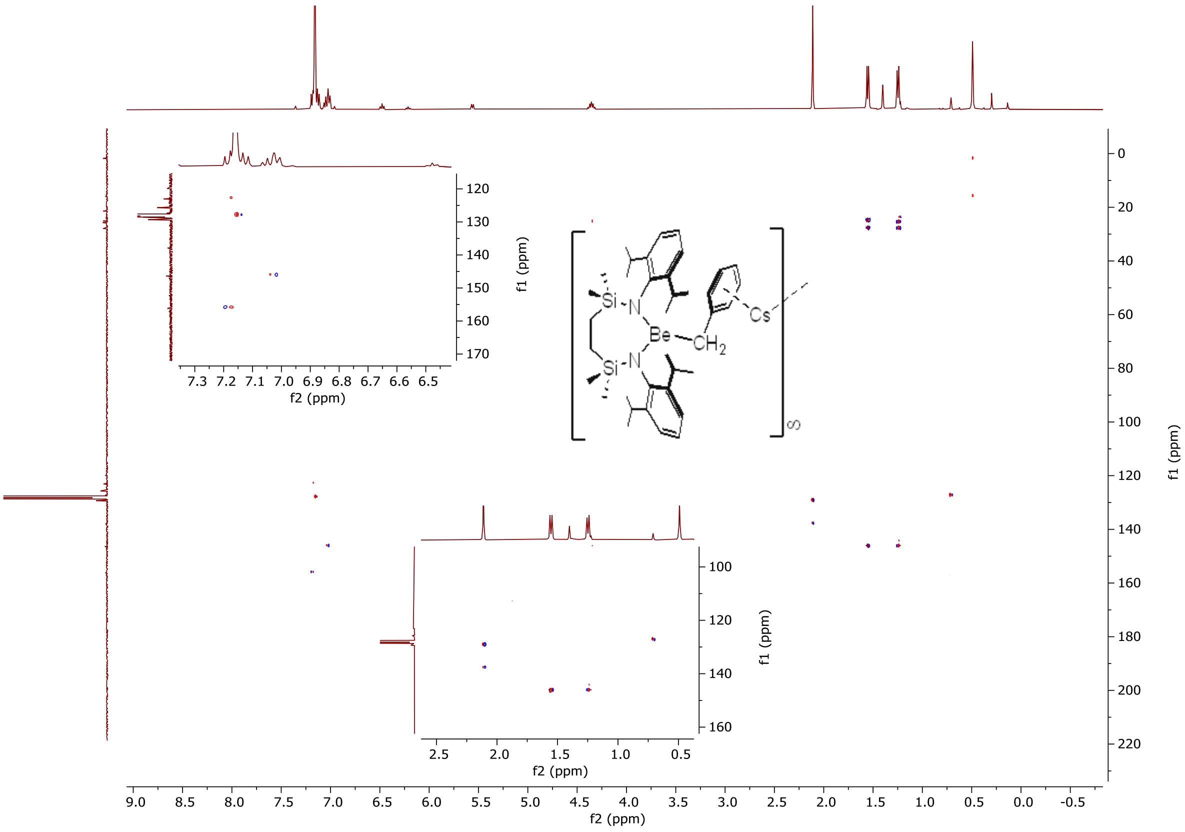

Figure S18. *^{1}H-^{13}C HMBC trace (C_6D_6, 298 K, 400.13, 100.62 MHz) for* ***[Cs({SiN^{Dipp}}BeCH$_2$C$_6$H$_5$)]$_\infty$ (3-h).***

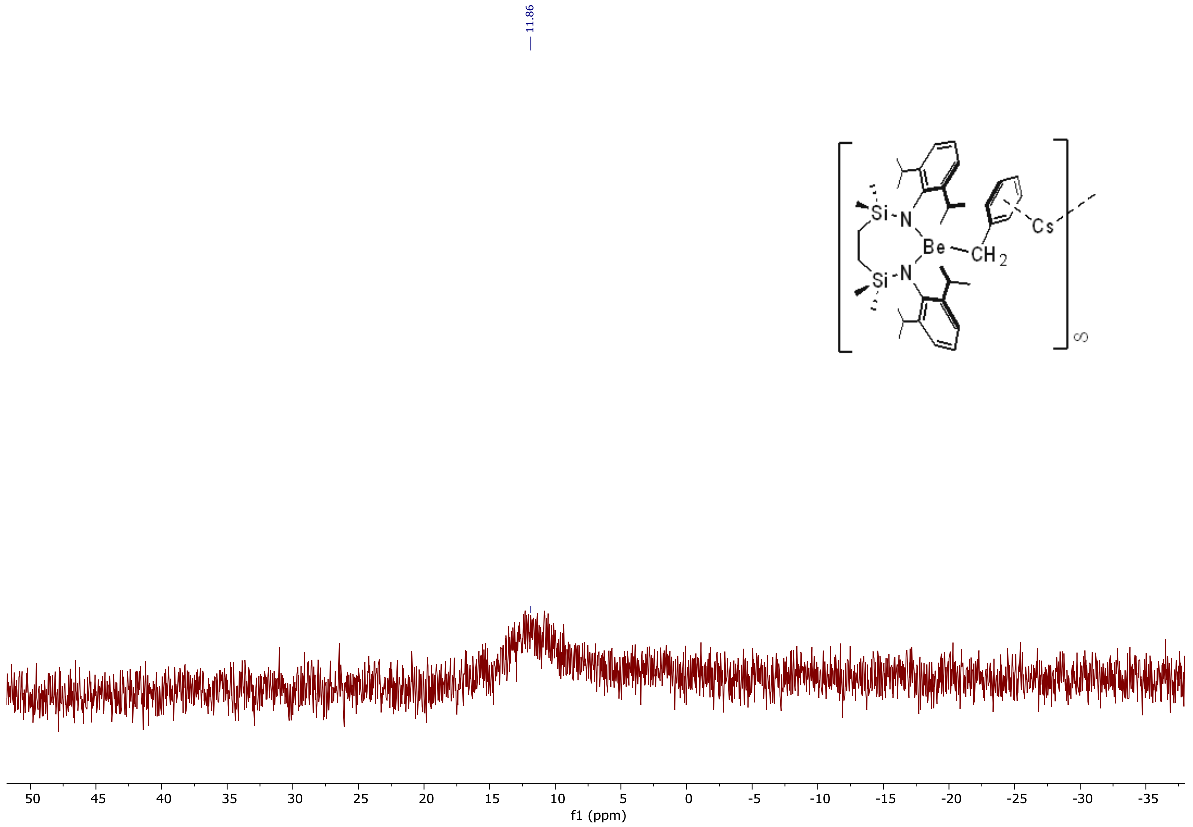

Figure S19. *[9]Be NMR Spectrum (C_6D_6, 298 K, 56.2 MHz) for* ***[Cs({SiN^{Dipp}}BeCH$_2$C$_6$H$_5$)]$_\infty$ (3-h)*****.**

Crystallographic Details

Single Crystal X–ray diffraction data for compounds **1, 2** and **3-*d*** were collected on a SuperNova, EosS2 diffractometer using CuKα (λ = 1.54184 Å) radiation throughout. The crystals were maintained at 150 K during data collection. Using Olex2,[3] the structures were solved with ShelXT and refined with the ShelXL[5] refinement package. Noteworthy points follow.

There is half of a dimer and half of a benzene molecule in the asymmetric unit of **1**. The remainders of both moieties arise *via* crystallographic inversion symmetry. H1 was located and its location was freely refined, while the associated U_{iso} was treated as riding on Cs1. H3 was also located and refined, in this case, at a distance of 0.98 Å from the parent atom. The U_{iso} values for any hydrogens which have the potential to be involved in interactions with the caesium centres (*i.e.* those attached to C13, C16, C27, C30, C38 and C39) were refined without restraints, although the hydrogens themselves were included at calculated positions. The sample was a 3-component twin, and this was addressed at the point of raw-data integration.

The asymmetric unit in **2** contains two dimers and a region of solvent that equates to one benzene molecule. The latter was disordered over at least three proximate sites but, even when this was modelled, there was evidence of significant correlation pointing to the fact that the proposal for the solvent region was, at best, an approximation. The observed electron-density smearing in this region was not altogether surprising as the solvent is located, edge-on, in narrow channels along the *b*-axis. Ultimately, however, the guest benzene was treated with the solvent mask algorithm available in Olex-2, and allowance made for same in the formula as presented. The beryllium-bound hydrogens were located and refined freely.

The crystal structure of **3-*d*** provides a very credible chemical characterisation of this beryllium and cesium-based, 2-D, polymeric structure. This was achieved in spite of sample handling difficulties (stability of material) and the twinned nature of the crystal. The latter was accounted for at the integration stage by treating the diffraction pattern as a 2-component twin, but there was also evidence of another (albeit very weakly diffracting) passenger present, which was ignored. Disorder in the asymmetric unit was confined to C15/C16 (55:45) and the toluene ligands based on C75 (63:37) and C85 (50:50). Disordered rings were treated as rigid hexagons and both distance and ADP restraints were employed, on merit, in all disordered regions to assist convergence.

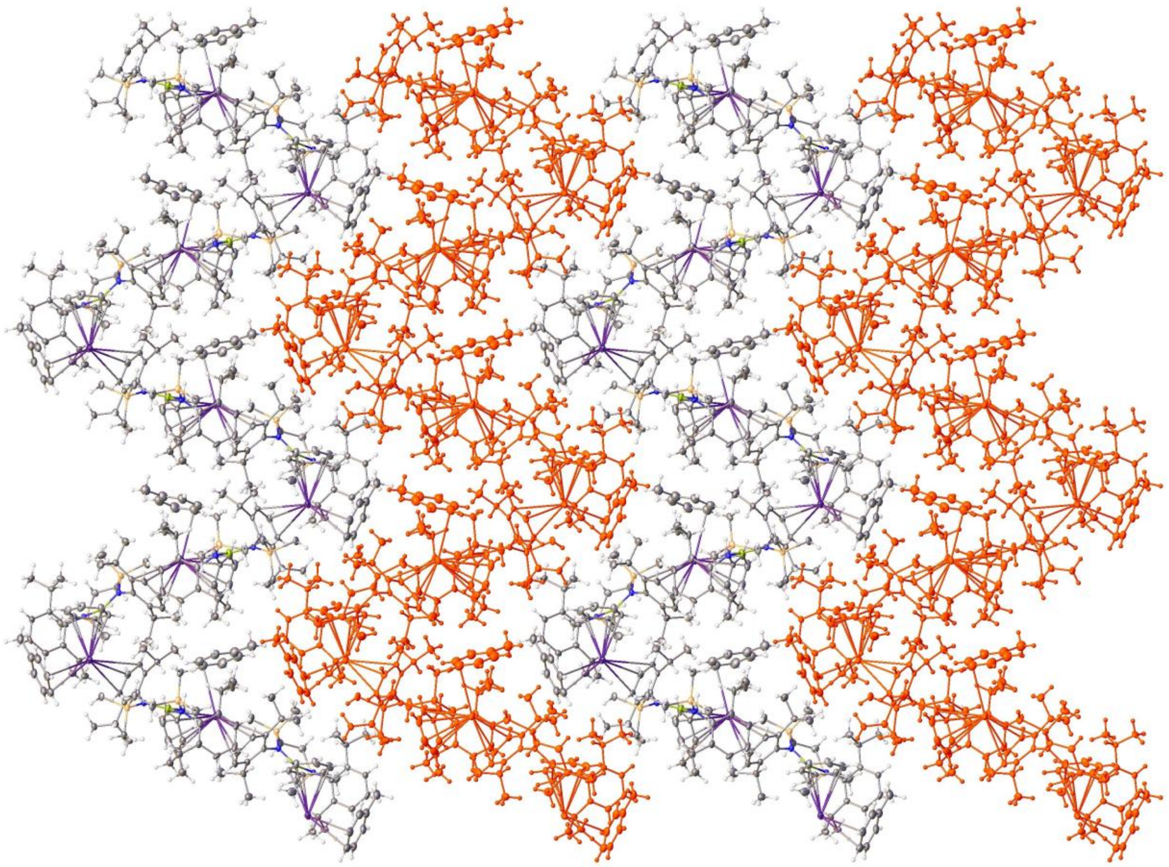

Figure S20. *Illustration down the b axis of the sheet structure formed by the packing of the 1-dimensional helical polymeric structure of **3-d**.*

Table S1: Crystal data and structure refinement for compounds **1**, **2** and **3-*d***.

Compound	**1**	**2**	**3-*d***
Empirical formula	$C_{51}H_{77}BeCs_2N_3Si_2$	$C_{63}H_{105}Be_2Cs_2N_4Si_4$	$C_{88}H_{130}Be_2Cs_2N_4Si_4$
Formula weight	1063.16	1314.70	1640.15
Crystal system	monoclinic	monoclinic	orthorhombic
Space group	$P2_1/n$	$P2_1/c$	$Pca2_1$
a/Å	10.50821(15)	41.46004(17)	33.9396(8)
b/Å	19.9949(2)	16.64434(8)	12.6657(4)
c/Å	25.9413(3)	20.71208(12)	20.6141(7)
α/°	90	90	90
β/°	91.4727(11)	97.0788(4)	90
γ/°	90	90	90
Volume/Å^3	5448.73(11)	14183.94(12)	8861.4(5)
Z	4	8	4
ρcalc g/cm3	1.296	1.231	1.229
μ/mm^{-1}	11.093	8.931	7.250
F(000)	2184.0	5480.0	3440.0
Crystal size/mm^3	0.062 × 0.051 × 0.039	0.1 × 0.1 × 0.1	0.116 × 0.081 × 0.058
2θ range /°	6.818 to 146.656	6.834 to 145.402	6.746 to 146.716
Index ranges	-12 ≤ h ≤ 13,<br>-22 ≤ k ≤ 24,<br>-32 ≤ l ≤ 30	-51 ≤ h ≤ 49,<br>-20 ≤ k ≤ 20,<br>-23 ≤ l ≤ 25	-41 ≤ h ≤ 39,<br>-15 ≤ k ≤ 15,<br>-25 ≤ l ≤ 25
Reflections collected	16770	188625	19170
Independent reflections [R_{int}, R_{sigma}]	16770 [R_{int} = 0.0471*, R_{sigma} = 0.0772]	28109 [R_{int} = 0.0364, R_{sigma} = 0.0233]	19170 [R_{int} = 0.1127*, R_{sigma} = 0.0955]
Data/restraints/parameters	16770/1/575	28109/0/1361	19170/609/1028
Goodness−of−fit on F^2	0.820	1.034	1.028
Final R indexes [I>=2σ (I)]	R_1 = 0.0329, wR_2 = 0.0573	R_1 = 0.0300, wR_2 = 0.0768	R_1 = 0.0747, wR_2 = 0.1929
Final R indexes [all data]	R_1 = 0.0575, wR_2 = 0.0610	R_1 = 0.0315, wR_2 = 0.0779	R_1 = 0.1082, wR_2 = 0.2094
Largest diff. peak/hole (e Å^{-3-})	0.60/-0.60	0.82/-0.80	0.67/-0.77
Flack parameter	–	–	-0.398(6)

*R_{int} pertaining to HKLF5 file (based on comparison of scale factors arising from the component analysis for each domain and overlapping reflections which contribute to each twinned component).

References

1. Dolomanov, O. V.; Bourhis, L. J.; Gildea, R. J.; Howard, J. A. K.; Puschmann, H., OLEX2: a complete structure solution, refinement and analysis program. *J. Appl. Cryst.* **2009**, *42*, 339-341.
2. Sheldrick, G. M., SHELXT - integrated space-group and crystal structure determination. *Acta Cryst*. **2015**, *A71*, 3-8.
3. Sheldrick, G. M., Crystal structure refinement with SHELXL. *Acta Cryst*. **2015**, *C71*, 3-8.
